# Are postoperative NLR and PLR associated with the magnitude of surgery-related trauma in young and middle-aged patients with bicondylar tibial plateau fractures? A retrospective study

**DOI:** 10.1186/s12891-021-04695-7

**Published:** 2021-09-23

**Authors:** Zhongzheng Wang, Yanwei Wang, Yuchuan Wang, Wei Chen, Yingze Zhang

**Affiliations:** 1 Department of Orthopaedic Surgery, Third Hospital of Hebei Medical University, 050051 Shijiazhuang, Hebei People’s Republic of China; 2 Key Laboratory of Biomechanics of Hebei Province, 050051 Shijiazhuang, Hebei People’s Republic of China; 3 Department of Orthopaedic Surgery, North China Medical and Health Group Xingtai General Hospital, 054000 Xingtai, Hebei People’s Republic of China; 4 NHC Key Laboratory of Intelligent Orthopaedic Equipment, 050051 Shijiazhuang, Hebei People’s Republic of China

**Keywords:** Neutrophil-to-Lymphocyte Ratio, Platelet-to-Lymphocyte Ratio, Surgery-related trauma, Bicondylar tibial plateau fractures

## Abstract

**Background:**

The invasiveness of different surgical procedures is variable. The purpose of this study was to investigate the value of the postoperative neutrophil-to-lymphocyte ratio (NLR) and platelet-to-lymphocyte ratio (PLR) as biomarkers in assessing the magnitude of surgery-related trauma in young and middle-aged patients with bicondylar tibial plateau fractures (TPFs).

**Methods:**

A total of 136 young and middle-aged patients with bicondylar TPFs who underwent surgical treatment between May 2016 and April 2020 were included. Details about demographic information, pre- and postoperative laboratory data, and surgical variables were obtained from the electronic database of our level I trauma center. According to the different surgery programs, all patients were divided into two groups: group 1, which represented minimally invasive reduction and internal fixation (MIRIF), and group 2, which represented open reduction and internal fixation (ORIF). Univariate and multivariate logistic regression and ROC curve analyses were used.

**Results:**

The operative time, intraoperative tourniquet use, intraoperative blood loss, length of incision, postoperative NLR, PLR, RBC and HCRP were significantly different between the two groups (*P* < 0.05). In the multivariate analysis, postoperative PLR ≥ 223.9, surgical incision > 19.0 cm and operative time > 130 min were closely related to severe surgery-related trauma. The ROC curve analysis indicated that postoperative PLR could predict severe surgery-related trauma with a specificity of 76.0 % and a sensitivity of 55.7 %.

**Conclusions:**

Postoperative PLR appears to be a useful biomarker that is closely associated with magnitude of surgery-related trauma in young and middle-aged patients with bicondylar TPFs.

## Background

Bicondylar tibial plateau fracture (TPF) management remains one of the most challenging orthopedic traumas, mainly occurring in young and middle-aged adults [1]. Bicondylar TPFs are often defined as types V and VI by the Schatzker classification system and 41-type C1-3 by AO/Orthopedic [2, 3]. Previous studies suggested that traditional open reduction and internal fixation (ORIF) treatment for patients with bicondylar TPFs represents a more invasive treatment with a higher risk of complications than minimally invasive reduction and internal fixation (MIRIF) [4, 5]. In the past few decades, many authors have proposed several minimally invasive surgery programs for bicondylar TPFs and have investigated their advantages through related studies [6, 7]. Several routine intra- and postoperative indicators, such as incision size, intraoperative blood loss and fluoroscopy frequency, operative time, postoperative complication rate, and healing time, have been successfully and widely used in assessing the invasiveness of a certain surgical procedure [8]. However, these parameters or indicators can only be roughly recorded or measured. Are there specific biomarkers that can more quickly and accurately reflect the invasiveness caused by surgery to patients? Which is worthy of our serious consideration.

The peripheral blood neutrophil-to-lymphocyte ratio (NLR) and platelet-to-lymphocyte ratio (PLR) have been increasingly recognized as indicators of a systematic inflammatory response for many malignant tumors, cardiovascular disorders, fractures and polytrauma [9–12]. A great deal of evidence suggests that severe trauma could trigger postoperative immune responses at the white blood cell levels, characterized by increasing neutrophil counts and decreasing numbers of lymphocytes [13, 14]. In addition, major trauma usually leads to increased platelet (PLT) activation and function, triggering the coagulation cascade and immune responses [15]. Recently, the NLR and PLR, calculated by dividing the neutrophil and platelet count by the lymphocyte count, have been considered simple, inexpensive and specific biomarkers of the immune response that can be easily obtained at most hospital laboratories. To our knowledge, there is no information about the possible use of NLR and PLR after surgery as predictors in assessing the invasiveness of bicondylar TPF surgical procedures.

Therefore, we performed a retrospective study to analyze the correlation between postoperative NLR and PLR levels and the magnitude of surgery-related trauma in young and middle-aged patients with bicondylar TPFs.

## Methods

### Study design and participants

**Fig. 1:**
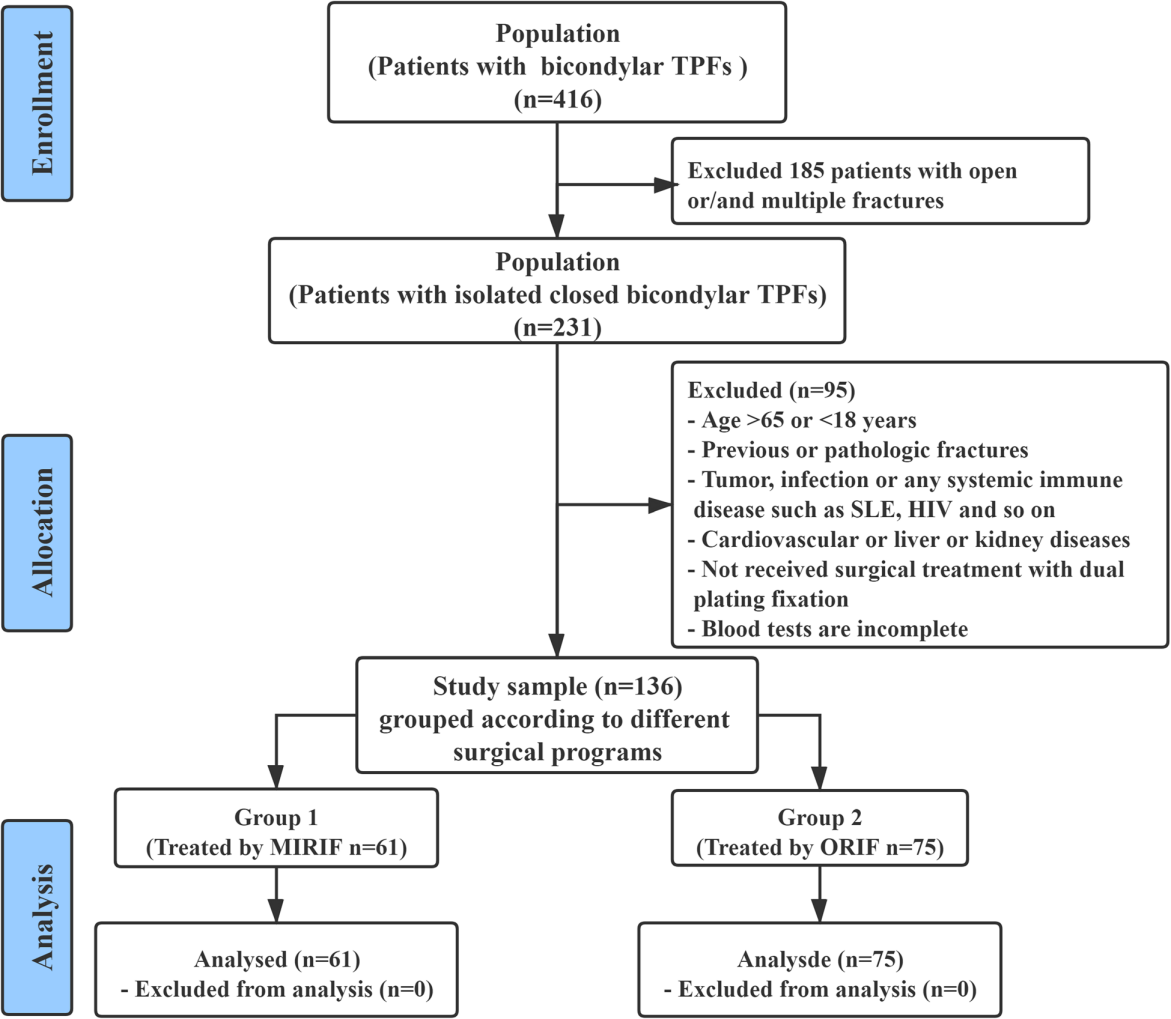
CONSORT flow diagram: selection of the study population

Data used in this study were extracted from an electronic database of our level I trauma center, in which a retrospective method was used to collect data on patients with bicondylar TPFs who underwent treatment with dual plating fixation (surgical procedures of MIRIF or ORIF) between May 2016 and April 2020. In this study, the inclusion criteria were defined as follows: (a) age ≥ 18 and ≤ 65 years; (b) diagnosis of closed bicondylar TPF; (c) surgical treatment with dual plating fixation; and (d) blood tests performed at hospital admission and on postoperative day 1. The exclusion criteria were as follows: (a) open wound, multiple trauma or compartment syndrome; (b) previous or pathologic fractures or old fractures (> 21 days); (c) active malignancy, systemic inflammatory or infectious diseases, systemic lupus erythematosus, HIV, severe neurovascular injury, or cardiovascular diseases; (d) treatment with external fixation or conservative methods; and (e) incomplete medical data. In this study, all patients with bicondylar TPFs were fixed by dual plating fixation. The patients were grouped according to the different surgical programs as follows: group 1 (treated by MIRIF, *n* = 61) and group 2 (treated by ORIF, *n* = 75). The flowchart of the patient screening process is shown in Fig. 1.

This study was reviewed and approved by our institutional ethics committee (entry no. 2015-003-1) and adhered to the principles outlined in the Helsinki Declaration. Informed consent was obtained from all patients in the study.

### Data collection of variables

The indicators of interest were extracted mainly as follows: (1) Demographic data such as age, sex, body mass index (BMI), lifestyle risk factors (smoking, alcoholism and living area), and chronic diseases (hypertension, diabetes); (2) Characteristics of fractures, including injury mechanism (low- or high-energy) and fracture classification; (3) Surgery-related variables included preoperative stay, surgical program (MIRIF and ORIF), operative time, intraoperative blood loss, anesthetization, American Society of Anesthesiologists (ASA) grade, and hospital stay; (4) Pre- and postoperative laboratory tests included neutrophil (N) count, lymphocyte (L) count, platelet (PLT) count, hypersensitive C-reactive protein (HCRP), hemoglobin (HGB), white blood cell (WBC), red blood cell (RBC) and others.

It is worth mentioning that the peripheral venous blood of patients was drawn by professionally trained phlebotomists. To control for circadian rhythms, all samples were routinely collected during the same time period (6:00 AM to 8:00 AM) on the1st day at admission and on the 1st postoperative day. The samples were transported to the biochemical laboratory for testing within an hour.

### Surgical procedures

**Fig. 2:**
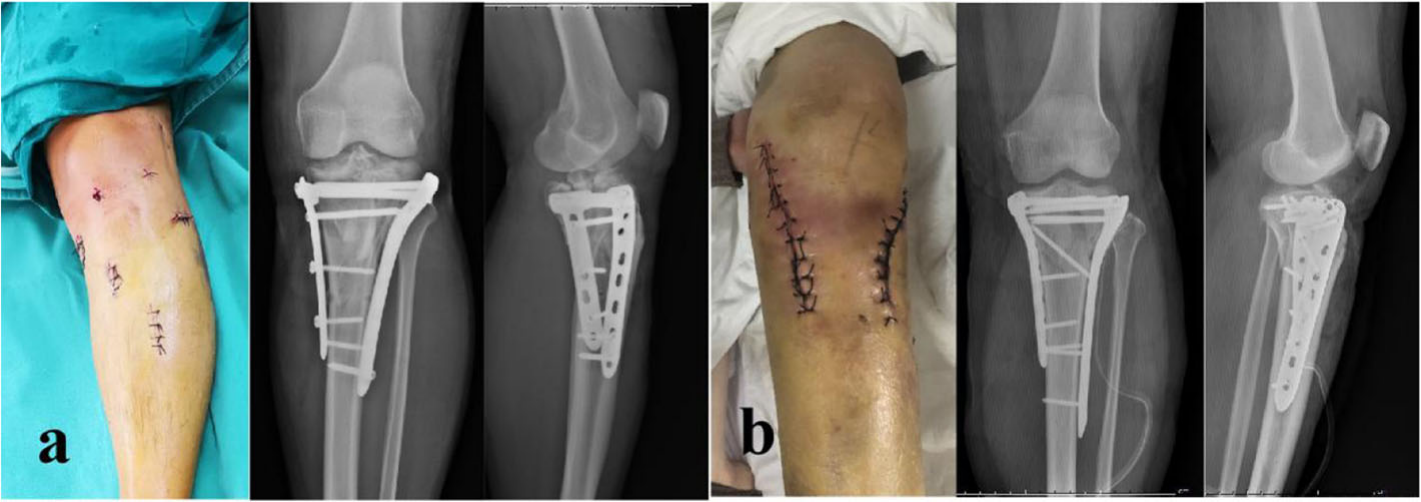
Surgical incision photos and immediate postoperative X-ray (AP and lateral views) of the two surgical programs. (**A**) Group 1 treated by MIRIF; (**B**) Group 2 treated by ORIF

Surgical procedures for the patients with bicondylar TPFs were performed by orthopedic surgeons from the same research team, and all surgeons had more than 10 years of experience in orthopedic surgery. Our team performed two different surgical procedures (MIRIF and ORIF) for patients with closed bicondylar TPFs. Before surgery, patients received general anesthesia or intraspinal anesthesia and routine antibiotic prophylaxis. The surgical program of group 1 was as follows: a universal traction device was used for bone traction of the lower extremities. Specially designed jacking tools were used to reduce the collapsed articular fragments. Intraoperative fluoroscopy was used to examine the reduction status. Two locking plates were placed on the medial and anterolateral tibial plateau, and the minimally invasive percutaneous plate osteosynthesis (MIPPO) technique was applied through 4–5 small incisions of approximately 3 cm [16] **(**Fig. 2A**)**. The surgical program of group 2 was as follows: two longitudinal incisions of approximately 10–15 cm were made on the medial and anterolateral tibial plateau, and two locking plates were inserted after prying reduction by Kirschner wires **(**Fig. 2B**)**. The other steps were the same as in group 1. In addition, postoperative care of the two groups of patients was standardized and kept uniform.

### Statistical analysis

All statistical data in our study were analyzed with IBM SPSS version 23.0 (IBM Armonk, New York, USA). Continuous variables are presented as the mean ± standard deviation (SD) as appropriate, and categorical variables are presented as numbers and percentages (%). According to whether the variables conformed to a normal distribution, the independent samples Student’s *t-test* or Mann–Whitney *U* test was used for continuous variables. Categorical variables were compared with the chi-square or Fisher exact test, as appropriate, to assess the significance of intergroup differences. In addition, the cutoff values, areas under the curve (AUCs) with 95 % confidence intervals (CIs), sensitivity and specificity of the relevant variables were analyzed using the receiver operating characteristic (ROC) curve procedure. Data with skewed distributions were categorized by the cutoff and reference values. Variables with significant differences (*P* < 0.05) were entered into multiple logistic regression analysis to determine the predictive factors for the magnitude of surgery-related trauma. The strength of the associations was evaluated according to the odds ratios (ORs) with 95 % CIs. The fitting degree of the final model was assessed by the Hosmer-Lemeshow test, and *P* > 0.05 indicated an acceptable result. A *P* value of less than 0.05 was considered statistically significant.

## Results

During a 4-year study period, a total of 136 patients with isolated bicondylar TPFs were recruited (71.3 % male; mean age 45.6 ± 10.8 years, 81.6 % high-energy injury). All patients were treated with dual plating fixation by one of two surgical procedures. The patients were divided into 2 subgroups by surgical procedure: group 1 included 61 patients (44.9 %) who underwent MIRIF, and group 2 consisted of 75 patients (55.1 %) who underwent ORIF.

**Fig. 3:**
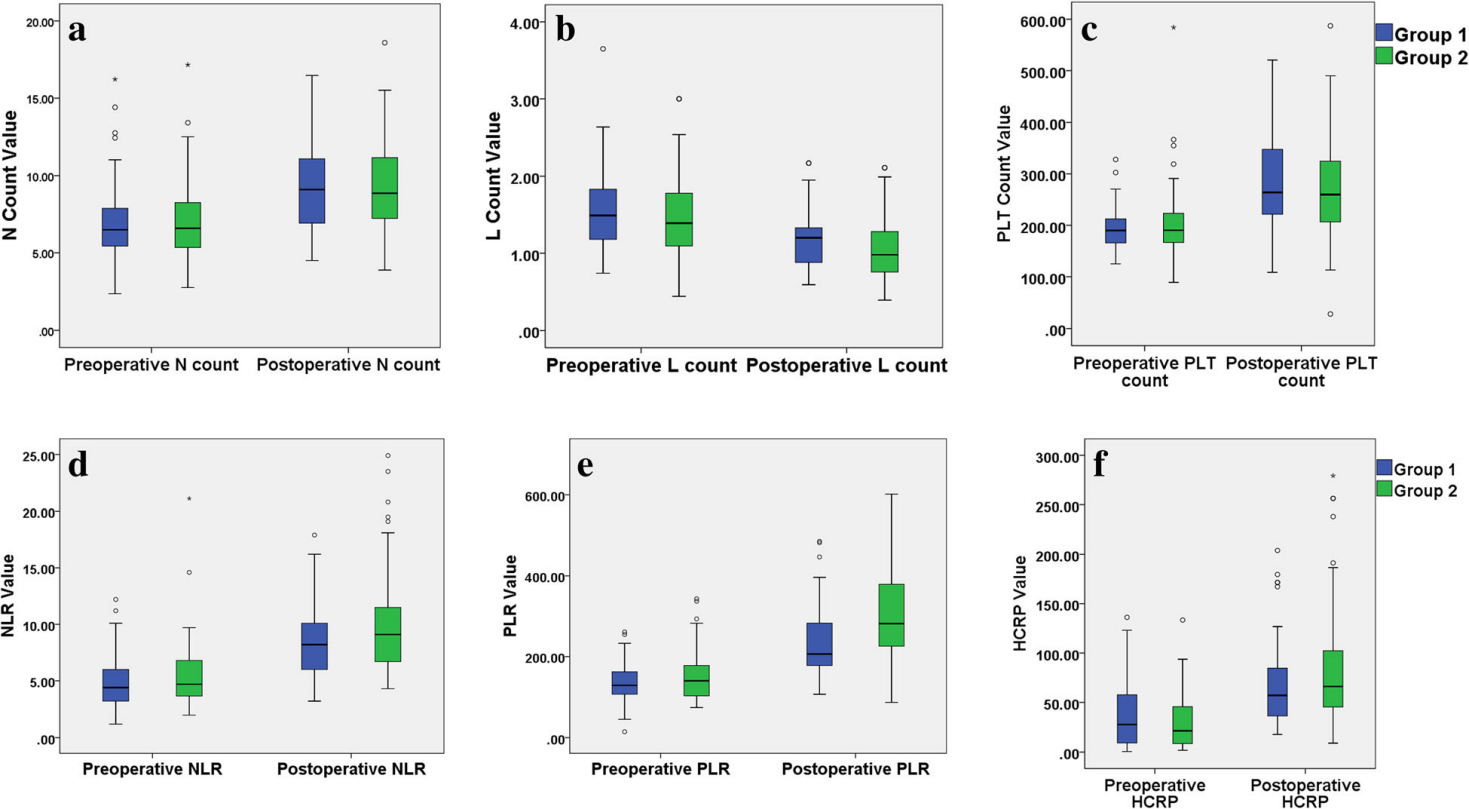
(**A**) neutrophil counts, (**B**) lymphocyte counts, (**C**) platelet counts, (**D**) neutrophil-to-lymphocyte ratio, (**E**) platelet-to-lymphocyte ratio and (**F**) hypersensitive C-reactive protein at admission and day 1 after surgery of the patients of two groups (boxplots with median, 75th and 90th percentile). Hollow circles represent values out of 90th percentile; Extreme values are shown as asterisks; Red double-tailed arrows indicate statistically significant difference between the two groups (*P* < 0.05)

In our study, the optimum cutoff values of related variables, such as the length of incision, operative time, intraoperative blood loss, pre- and postoperative NLR, PLR and HCRP, were determined by ROC curve analysis (Table 1). In the univariate analysis, there was no significant differences in the baseline characteristics of the patients or preoperative hematologic indicators between the two groups. The operative time (*P* < 0.001), intraoperative tourniquet use (*P* = 0.015), intraoperative blood loss (*P* < 0.001), length of incision (*P* < 0.001), postoperative 1st day NLR > 10.1 (*P* = 0.018), postoperative 1st day PLR > 223.9 (*P* < 0.001), postoperative 1st day RBC < lower limit (*P* = 0.021) and postoperative 1st day HCRP > 47.6 mg/L (*P* = 0.048) of patients in group 1 were significantly different from those in group 2. In addition, pre- and postoperative laboratory findings showed that postoperative RBC, HGB, and ALB were significantly lower than their preoperative values, and postoperative N count, PLT count, L count, NLR, PLR and HCRP were significantly higher than those before surgery **(**Table 2 and Fig. 3**)**.

**Table 1:** Optimum cut-off value of continuous variables detected by the ROC cutve analysis

Variables	Cut-off value	Area under the ROC curve (AUC)	95 % CI	P-value
Preoperative stay (days)	9.0	0.54	0.44–0.63	0.468
Length of incision (cm)	19.0	0.85	0.78–0.91	< 0.001
Operative time (min)	130.0	0.85	0.79–0.92	< 0.001
Preoperative NLR	5.9	0.55	0.45–0.65	0.338
Preoperative PLR	137.5	0.58	0.48–0.67	0.129
Preoperative HCRP	16.1	0.47	0.37–0.56	0.480
Postoperative 1st day NLR	10.1	0.58	0.49–0.68	0.095
Postoperative 1st day PLR	223.9	0.68	0.59–0.77	< 0.001
Postoperative 1st day HCRP	47.6	0.56	0.51–0.70	0.225

Note: *P* value < 0.05 was considered statistically significant. Abbreviations: ROC, receiver operating characteristic; AUC, areas under the curve; CI, confidence interval; NLR, neutrophil-to-lymphocyte ratio; PLR, platelet-to-lymphocyte ratio; HCRP, hypersensitive C-reactive protein

**Table 2:** Univariate analyses of variables associated with the magnitude of surgery-related trauma

Variable	Total patients (*n* = 136)	Group 1 (*n *= 61)	Group 2 (*n* = 75)	*P*
**Baseline characteristics of the patients**				
Sex (male), n (%)	97 (71.3)	47 (77.0)	50 (66.7)	0.183
Age (years), mean ± SD	45.6 ± 10.8	43.9 ± 10.7	47.1 ± 10.8	0.090
BMI (Kg/m^2), mean ± SD	26.2 ± 3.3	26.4 ± 3.2	26.0 ± 3.5	0.508
Tobacco consumption (yes), n (%)	31 (22.8)	17 (27.9)	14 (18.7)	0.203
Alcohol consumption (yes), n (%)	29 (21.3)	15 (24.6)	14 (18.7)	0.402
Hypertension (yes), n (%)	24 (17.6)	11 (18.0)	13 (17.3)	0.915
Diabetes (yes), n (%)	11 (8.1)	6 (9.8)	5 (6.7)	0.500
Living area, n (%) Rural Urban	87 (64.0) 49 (36.0)	35 (57.4) 26 (42.6)	52 (69.3) 23 (30.7)	0.149
Injury mechanisms (high energy), n (%)	111 (81.6)	48 (78.7)	63 (84.0)	0.426
Schatzker classification, n (%) Type V Type VI	68 (50.0) 68 (50.0)	29 (47.5) 32 (52.5)	39 (52.0) 36 (48.0)	0.605
Preoperative stay (days), n (%) 0–9 cutoff ≥ 10	106 (77.9) 30 (22.1)	51 (83.6) 10 (16.4)	55 (73.3) 20 (26.7)	0.151
**Preoperative hematologic indicators**				
Neutrophil count (> 6.30 × 10^9/L, reference), n (%)	77 (56.6)	35 (57.4)	42 (56.0)	0.872
Lymphocyte count (< 1.10 × 10^9/L, reference), n (%)	28 (20.1)	9 (14.7)	19 (25.3)	0.129
PLT count (× 10^9/L), n (%) 125–350 reference < 125 > 350	127 (93.4) 5 (3.7) 4 (2.9)	58 (95.1) 2 (3.3) 1 (1.6)	69 (92.0) 3 (4.0) 3 (4.0)	0.698
AST/ALT (> 1, reference), n (%)	67 (49.3)	31 (50.8)	36 (48.0)	0.744
WBC (> 10 × 10^9/L, reference), n (%)	44 (32.4)	17 (27.9)	27 (36.0)	0.313
NLR (> 5.9, cutoff), n (%)	45 (33.1)	16 (26.2)	29 (38.7)	0.125
PLR (> 137.5, cutoff), n (%)	68 (50.0)	28 (45.9)	40 (53.3)	0.389
ASA class, n (%) I II III or above	13 (9.6) 110 (80.8) 13 (9.6)	7 (11.5) 51 (83.6) 3 (4.9)	6 (8.0) 59 (78.7) 10 (13.3)	0.221
RBC (< lower limit × 10^12/L, reference), n (%)	53 (39.0)	22 (36.1)	31 (41.3)	0.531
HGB (< lower limit × g/L, reference), n (%)	78 (57.4)	31 (50.8)	47 (62.7)	0.165
ALB (< 40 g/L, reference), n (%)	49 (36.0)	20 (32.8)	29 (38.7)	0.477
HCRP (> 16.1 mg/L, cutoff), n (%)	82 (60.3)	35 (57.4)	47 (62.7)	0.531
**Intraoperative indexes**				
Anesthetization, n (%) General Intraspinal	87 (64.0) 49 (36.0)	44 (68.9) 17 (31.1)	45 (60.0) 30 (40.0)	0.285
Bone grafting (yes), n (%)	62 (45.6)	31 (50.8)	31 (41.3)	0.269
Operative time (minutes), n (%) 0-130 cutoff > 130	61 (44.9) 75 (55.1)	45 (73.8) 16 (26.2)	16 (21.3) 59 (78.7)	< 0.001
Intraoperative tourniquet use (yes), n (%)	78 (57.4)	28 (45.9)	50 (66.7)	0.015
Intraoperative blood loss (> 330ml, cutoff), n (%)	54 (39.7)	13 (21.3)	41 (54.7)	< 0.001
Length of incision (cm), n (%) 0–19 cutoff > 19.0	64 (47.1) 72 (52.9)	50 (82.0) 11 (18.0)	14 (18.7) 61 (81.3)	< 0.001
**Hematologic indexes of the postoperative 1st day**				
Neutrophil count (> 6.30 × 10^9/L, reference), n (%)	119 (87.5)	50 (82.0)	69 (92.0)	0.078
Lymphocyte count (< 1.10 × 10^9/L, reference), n (%)	72 (52.9)	27 (44.3)	45 (60.0)	0.067
PLT count (× 10^9/L), n (%) 125–350 reference < 125 > 350	105 (77.2) 5 (3.7) 26 (19.1)	45 (73.8) 4 (6.6) 12 (19.6)	60 (80.0) 1 (1.3) 14 (18.7)	0.261
WBC (> 10 × 10^9/L, reference), n (%)	88 (64.7)	38 (62.3)	50 (66.7)	0.596
NLR (> 10.1, cutoff), n (%)	48 (35.3)	15 (24.6)	33 (44.0)	0.018
PLR (> 223.9, cutoff), n (%)	83 (61.0)	27 (44.3)	56 (74.7)	0.001
RBC (< lower limit × 10^12/L, reference), n (%)	106 (77.9)	42 (68.9)	64 (85.3)	0.021
HGB (< lower limit × g/L, reference), n (%)	113 (83.1)	46 (77.0)	67 (88.0)	0.090
ALB (< 35 g/L, reference), n (%)	72 (52.9)	30 (49.2)	42 (56.0)	0.428
HCRP (> 47.6 mg/L, cutoff), n (%)	88 (64.7)	34 (55.7)	54 (72.0)	0.048

Note: *P* value < 0.05 was considered statistically significant. Cutoff values are determined by ROC analysis. Abnormal reference range for RBC: male < 4 × 10^12/L and female < 3.5 × 10^12/L, for HGB: male < 130 g/L and female < 115 g/L. Abbreviations: BMI, body mass index; PLT, platelet; AST, aspartate aminotransferase; ALT, alanine transaminase; WBC, white blood cell; NLR, neutrophil-to-lymphocyte ratio; PLR, platelet-to-lymphocyte ratio; ASA, American Society of Anesthesiologists; RBC, red blood cell; HGB, hemoglobin; ALB, albumin; HCRP, hypersensitive C-reactive protein

**Fig. 4:**
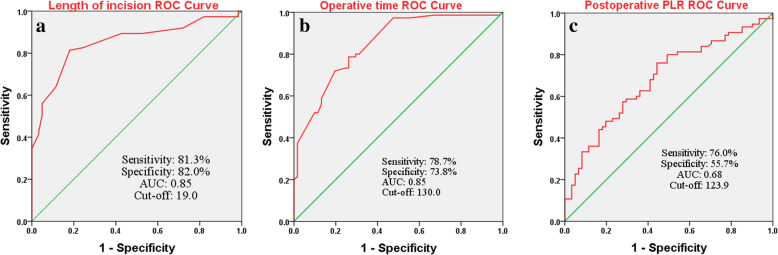
ROC curve analysis was performed to determine cut-off value of **A** (the incision length), **B** (operative time) and **C** (postoperative PLR), and to calculate the sensitivity, specificity and AUC (area under curve) for predicting the severity of surgery-related trauma

In the multivariate logistic regression analysis model, some related factors, such as age, sex, BMI, hypertension, diabetes, anesthetization and postoperative 1st day HGB, were included, which may influence the choice of surgical procedures and the body’s immune response. The final multivariate analysis results suggested that incision > 19.0 cm (OR 14.71, 95 % CI 4.03–52.63, *P* < 0.001), operative time > 130 min (OR 6.94, 95 % CI 2.32–21.27, *P* = 0.001) and postoperative 1st day PLR > 223.9 (OR 4.24, 95 % CI 1.20-14.93, *P* = 0.025) had a significant association with severe surgery-related trauma **(**Table 3**)**. The Hosmer-Lemeshow test showed the good fitness (*X*^*2*^ = 10.858, *P* = 0.21, Nagelkerke R^2^ = 0.663). We further constructed ROC curves and used the AUC, sensitivity, and specificity to evaluate the predictive ability of each biomarker or related index: length of incision (cutoff 19 cm, AUC: 0.85, sensitivity: 81.3 %, specificity, 82.0 %, *P* < 0.001), operative time (cutoff 130 min, AUC: 0.85, sensitivity: 78.7 %, specificity, 73.8 %, *P* < 0.001) and postoperative 1st day PLR (cutoff 223.9, AUC: 0.68, sensitivity: 76.0 %, specificity, 55.7 %, < 0.001) **(**Fig. 4**)**.

**Table 3:** Multivariable logistic regression analyses of variables associated with the magnitude of surgery-related trauma

Variable	Multivariate analysis		*P*
	**OR**	**95 % CI**	
Length of incision (> 19.0 cm)	14.71	4.03–52.63	< 0.001
Operative time (> 130 min)	6.94	2.32–21.27	0.001
Postoperative 1st day PLR (> 223.9)	4.24	1.20-14.93	0.025
Postoperative 1st day NLR (> 10.1)	1.30	0.36–4.69	0.691
Postoperative 1st day RBC (< lower limit × 10^12/L)	1.19	0.20–7.02	0.846
Postoperative 1st day HCRP (> 47.6 mg/L)	2.11	0.64–6.99	0.218
Postoperative 1st day HGB (< lower limit × g/L)	2.44	0.27–22.22	0.429
Intraoperative tourniquet use (yes)	1.26	0.38–4.19	0.711
Intraoperative blood loss (> 330ml)	2.18	0.60-8.00	0.238
Sex (male)	1.93	0.56–6.71	0.297
Age (years)	1.01	0.95–1.07	0.699
BMI (Kg/m^2)	1.01	0.85–1.21	0.874
Hypertension (yes)	1.28	0.24–6.85	0.776
Diabetes (yes)	1.38	0.12–16.13	0.804
Anesthetization (general)	2.74	0.83–9.02	0.097

Note: *P* value < 0.05 was considered statistically significant. Abnormal reference range for RBC: male < 4 × 10^12/L and female < 3.5 × 10^12/L, for HGB: male < 130 g/L and female < 115 g/L. Abbreviations: BMI, body mass index; NLR, neutrophil-to-lymphocyte ratio; PLR, platelet-to-lymphocyte ratio; RBC, red blood cell; HGB, hemoglobin; HCRP, hypersensitive C-reactive protein

## Discussion

In recent years, many studies have found that the NLR and PLR are related to the prognosis of patients with severe trauma [11, 12, 14, 17]. To our knowledge, this is the first study to use the simple, inexpensive laboratory indicators NLR and PLR to assess the magnitude of surgery-related trauma in young and middle-aged patients with bicondylar TPFs. In our study, the results of multiple logistic regression analysis showed that the postoperative 1st day PLR > 223.9 (OR 4.24, 95 % CI 1.20-14.93, *P* = 0.025) was an independent predictor of severe surgery-related trauma in patients with isolated bicondylar TPFs.

In the past few years, minimally invasive treatment techniques such as MIPPO technology and double-reverse traction technology have been widely used in the treatment of bicondylar TPFs [7, 16]. Compared with traditional ORIF, the advantages of MIRIF are becoming increasingly obvious [4–7]. Assessing the severity of surgery-related trauma has always been an integral part of orthopedic trauma management. A number of intraoperative and postoperative indicators have been widely and successfully used in assessing the severity of the trauma caused by surgical procedures. Among them, the operation time, intraoperative blood loss, and incision size were the most commonly used indicators to assess the severity of surgical trauma [18, 19]. However, as far as I know, these indicators have not been precisely measured. Surgeons are still researching simple and credible biochemical parameters that can reflect the severity of surgery-related trauma.

Recently, several ready-made parameters, derived from routine complete blood counts (CBCs), have been investigated as potential biomarkers. In response to major trauma, neutrophils can rapidly contribute to the inflammatory response; as part of a systemic inflammatory response, lymphocytes are involved in the adaptive immune response. It is characterized by an increase in neutrophil counts and a decrease in lymphocyte counts. Platelets are also activated after traumatic injuries as cellular effectors of inflammation [20, 21]. Previous studies have shown that the NLR and PLR are prognostic markers for many diseases, such as cancer, cardiovascular disease, chronic inflammation, hip fractures and polytrauma [9–12, 17]. In our early study, it was found that the NLR level within 48 h after injury was a useful biomarker for predicting the severity of TPF injury, which was the first study of the relationship between the NLR and the severity of a fracture [11]. Wasko et al. [14] indicated that postoperative NLR showed faster changing kinetics than C-reactive protein in response to the surgical trauma of total hip arthroplasty and total knee arthroplasty. In addition, Pehlivanl et al. [22] indicated that PLR is valuable in the diagnosis and differentiation of acute appendicitis. However, there are still only limited data about the possible role of NLR and PLR in predicting the invasiveness of surgery procedures.

It is important to assess the severity of surgery-related trauma by identifying reliable biomarkers. Flohé et al. [23] reported that surgical trauma causes similar changes in the immune response to those observed after accidental injury. Surgical intervention is also an additional challenge to the immune system, which may be superimposed on the stronger immune response caused by trauma. The results of our study also confirmed this point. Postoperative inflammatory indexes, such as N counts, L counts, PLT counts, NLR, PLR and HCRP, had obvious superposition effects compared with primary trauma (Table 2 and Fig. 3). Pape et al. [24] suggested that the duration of surgery and the grade of surgery were important factors affecting the severity of postoperative complications. In addition, Decker et al. [25] investigated that laparoscopic surgery, a less invasive procedure, caused changes in the immune response (type-1/type-2 T-helper cell balance) compared to conventional approaches to cholecystectomy and verified whether changes in the Th1/Th2 balance were sensitive enough to quantify less invasive surgical interventions. Therefore, the author hypothesized that elevated postoperative NLR and PLR levels may be related to the severity of surgery-related trauma, and our analysis results also supported this hypothesis.

The results of univariate analysis showed that there were significant differences between the two groups in incision size, operative time, intraoperative blood loss, postoperative RBC and HGB (Table 2). This indirectly reflected that the ORIF surgical procedure had greater aggressiveness. Multivariate logistic regression analysis confirmed that the length of incision > 19.0 cm, operative time > 130 min and postoperative 1st day PLR > 223.9 were closely related to severe surgery-related trauma. Compared with postoperative NLR, postoperative PLR was more strongly associated with the severity of surgical-related trauma (OR: 4.24 vs. OR: 1.30) (Table 3). In our study, although the ROC analysis results showed that the AUC, sensitivity and specificity of postoperative PLR were lower than those of surgical incision length and operation time, it could still accurately reflect the postoperative immune status of patients.

Of note, the present study has some limitations. First, this study was a retrospective and single-center study. Second, this study failed to elaborate in detail on the pathogenesis of the elevated postoperative NLR and PLR levels caused by major surgery. Third, there is a lack of standardized management of postoperative hematology examinations in our center. Except for routine whole blood cell examination on the first day after surgery, there was no definite time for other blood examinations. Finally, the laboratory test results of certain clinical and inflammatory markers, such as interleukin (IL)-6, IL-10 and TNF-α, were not included in this study. Therefore, the results of this study should be verified in further prospective and multicenter studies.

## Conclusions

To our knowledge, this is the first study to clarify that postoperative NLR and PLR can be used to predict the magnitude of surgery-related trauma by restricting the fracture types and surgery programs. Postoperative 1st day PLR > 223.9, length of incision > 19.0 cm and operative time > 130 min were closely correlated with severe surgery-related trauma in young and middle-aged patients with bicondylar TPFs. The potential value of postoperative PLR in clinical practice needs to be established in additional studies.

## Data Availability

The datasets used and/or analyzed during the current study available from the corresponding author on reasonable request.

## Abbreviations

NLR: neutrophil-to-lymphocyte ratio
PLR: platelet-to-lymphocyte ratio
TPFs: tibial plateau fractures
MIRIF: minimally invasive reduction and internal fixation
ORIF: open reduction and internal fixation
PLT: platelet
BMI: body mass index
ASA: American Society of Anesthesiologists
HCRP: hypersensitive C-reactive protein
HGB: hemoglobin
ALB: albumin
WBC: white blood cell
RBC: red blood cell
MIPPO: minimally invasive percutaneous plate osteosynthesis
AUCs: areas under the curve
CIs: confidence intervals
ORs: odds ratios
ROC: receiver operating characteristic
