# Correction to: Are postoperative NLR and PLR associated with the magnitude of surgery-related trauma in young and middle-aged patients with bicondylar tibial plateau fractures? A retrospective study

**DOI:** 10.1186/s12891-022-04996-5

**Published:** 2022-01-17

**Authors:** Zhongzheng Wang, Yanwei Wang, Yuchuan Wang, Wei Chen, Yingze Zhang

**Affiliations:** 1 Department of Orthopaedic Surgery, Third Hospital of Hebei Medical University, 050051 Shijiazhuang, Hebei People’s Republic of China; 2 Key Laboratory of Biomechanics of Hebei Province, 050051 Shijiazhuang, Hebei People’s Republic of China; 3 Department of Orthopaedic Surgery, North China Medical and Health Group Xingtai General Hospital, 054000 Xingtai, Hebei People’s Republic of China; 4 NHC Key Laboratory of Intelligent Orthopaedic Equipment, 050051 Shijiazhuang, Hebei People’s Republic of China

**Correction to: BMC Musculoskelet Disord 22, 816 (2021)**

**https://doi.org/10.1186/s12891-021-04695-7**

Following the publication of the original article [1] the authors noticed that the note for equal contributors “†Zhongzheng Wang and Yanwei Wang contributed equally to this work.” did not appear in the proof.

The original article [1] has been updated.
